# Early Childhood Caries and Body Mass Index in Young Children from Low Income Families

**DOI:** 10.3390/ijerph10030867

**Published:** 2013-03-05

**Authors:** Luciane Rezende Costa, Anelise Daher, Maria Goretti Queiroz

**Affiliations:** 1 Department of Preventive and Restorative Dentistry, Faculty of Dentistry, Federal University of Goias, Primeira Avenida, Setor Universitario, Goiania, GO 74605-220, Brazil; 2 Health Sciences Graduate Program, Federal University of Goias, Primeira Avenida, Setor Universitario, Goiania, GO 74605-220, Brazil; E-Mail: anelisedaher@terra.com.br; 3 Department of Stomatological Sciences, Faculty of Dentistry, Federal University of Goias, Primeira Avenida, Setor Universitario, Goiania, GO 74605-220, Brazil; E-Mail: mgorettiq@gmail.com

**Keywords:** oral health, preschool children, body mass index, dental caries, socioeconomic status

## Abstract

The relationship between early childhood caries (ECC) and obesity is controversial. This cross-sectional survey investigated this association in children from low-income families in Goiania, Goias, Brazil and considered the role of several social determinants. A questionnaire examining the characteristics of the children and their families was administered to the primary caregiver during home visits. In addition, children (approximately 6 years of age) had their height, weight, and tooth condition assessed. The primary ECC outcome was categorized as one of the following: caries experience (decayed, missing, filled tooth: “dmft” index > 0), active ECC (decayed teeth > 0), or active severe ECC (decayed teeth ≥ 6). Descriptive, bivariate and logistic regression analyses were conducted. The participants in the current study consisted of 269 caregiver-child dyads, 88.5% of whom were included in the Family Health Program. Caregivers were mostly mothers (67.7%), were 35.3 ± 10.0 years old on average and had 9.8 ± 3.1 years of formal education. The mean family income was 2.3 ± 1.5 times greater than the Brazilian minimum wage. On average, the children in the current study were 68.7 ± 3.8 months old. Of these, 51.7% were boys, 23.4% were overweight or obese, 45.0% had active ECC, and 17.1% had severe ECC. The average body mass index (BMI) of the children was 15.9 ± 2.2, and their dmft index was 2.5 ± 3.2. BMI was not associated with any of the three categories of dental caries (*p* > 0.05). In contrast, higher family incomes were significantly associated with the lack of caries experience in children (OR 1.22, 95%CI 1.01–1.50), but the mother’s level of education was not significantly associated with ECC.

## 1. Introduction

Early childhood caries (ECC), which results from a chronic imbalance between multiple risk factors and protective factors, remains a public health problem in many communities [1]. A review by the American Academy of Pediatric Dentistry [1] highlighted investigations that have demonstrated the consequences of ECC on the development of new carious lesions in both primary and permanent dentitions, increased treatment costs, delayed physical growth and development, loss of school days, increased numbers of days with restricted activity and a diminished ability to learn. The negative impact of severe forms of ECC (S-ECC) on the quality of life of young children and their families has been documented in the literature [2,3,4] and can require dental rehabilitation under general anesthesia [5].

Because the global increase in childhood obesity throughout diverse populations shares several behavioral and social etiological factors with dental caries, multiple studies have attempted to explore the associations between these two chronic diseases in both primary and permanent dentitions, but the results of these studies remain controversial. In 2006, a systematic review concluded that only one study showed a direct association between obesity and dental caries with a high level of evidence [6]. Since then, several retrospective studies of primary dentitions have shown that obese and overweight children may have less tooth caries than children who are underweight or healthy weight [4,7]. However, other retrospective studies have suggested that there is no relationship between S-ECC and body mass index (BMI) [8]. Cross-sectional studies examining ECC risk have also reported conflicting results for underweight children [9,10,11,12,13,14,15,16] and overweight children [17,18,19,20,21,22,23,24,25]. BMI has been reported to exert a weak independent effect on caries variation in a cohort of Swedish children [26]. Similarly, longitudinal studies have come to conflicting conclusions regarding the benefits of dental ECC treatments for improving anthropometric measurements in children [2,10,27].

In addition to differences in study design, it is possible that the aforementioned conflicting results can be related to variations in the setting where data were collected, the socioeconomic status of the sample, the nutritional status measures employed, discrepancies in dental caries, the specific caries assessment index used and differences in the ages of the children examined, among other factors [14,15,16,23,25,26,28]. One prospective study reported that the association between caries prevalence and obesity increases with increasing age. Specifically, no significant association was observed at 3 years of age (odds ratio = 1.0), but a significant association was observed at age 6 (odds ratio = 2.5), with higher caries prevalence in overweight and obese adolescents (15 years) and young adults (20 years) [29]. In contrast, the American National Health and Nutrition Examination Survey 1999–2002 reported that being overweight may be associated with decreased rates of caries in older children [30].

Although the occurrence of ECC is decreasing in some countries [31], certain subpopulations of preschoolers still exhibit a high prevalence of ECC, even in developed countries, which shows the skewed distribution of this disease [10] and the need for a global effort to understand the factors associated with it. The aim of this study was to analyze the relationship between ECC and childhood BMI. In addition, the social determinants that can be associated with both caries and unsatisfactory BMI were investigated.

## 2. Methods

### 2.1. Participants

This cross-sectional survey was conducted in 2009–2010 with families living in the East and North Health Districts of Goiania in the state of Goias in Midwest Brazil. These districts are located in an urban area within the city of Goiania. A subset of the families examined here received assistance from the Family Health Program (FHP). The FHP’s family-focused multidisciplinary primary care is provided by teams of doctors, nurses, nursing assistants and community health agents, either at health centers or at the patient’s home. Many of these health teams also include dentists and dental assistants. The FHP teams are responsible for following approximately 1,000 families in a well-defined geographical area. The East Health District is divided into 15 public health units, and it has an estimated population of 177,661 (13.8% of the Goiania population as of 2009). This population includes 363 families with 6-year-old children and is served by 18 health family teams. The North Health District comprises 12 public health units with18 health family teams serving an estimated population of 142,251 (11.0% of the city’s population as of 2009), including 420 families with children who are approximately 6 years old. These health districts were chosen because they were already being used in the “Tutorial Education Program at Work” (PET-Saude), a government program supported by the Ministries of Health and Education that aims to increase the involvement of undergraduate students from the Federal University of Goias in community services.

This study focused on the child-primary caregiver dyad in families with children born in 2004. Children with any mental or physical disabilities that precluded the examination procedures and those who were uncooperative were excluded from the study. In addition, families who withdrew their consent to participate were also excluded from the final analysis.

It was estimated that a minimum sample size of 275 families was required to achieve a level of precision of 10% with an alpha of 5%. To perform this calculation, the prevalence of dental caries was considered to be 58.3%, based on a study of 5-year-old Brazilian children living in Midwest Brazil [32]. Following a probabilistic stratified sampling model, indexed families were divided into strata according to their area/micro-area, and the final sample was obtained through a simple random sampling of each stratum. We included a 10% excess of participants (based on power analysis) to overcome the potential loss of some participants during data collection.

Ethical approval was obtained in 2009 from the Institutional Research Board of the Federal University of Goias (UFG), Brazil (protocol #128/09). Informed consent was sought and obtained from all participants (*i.e.*, the parents on behalf of their children) after the aims, risks, and benefits of the study were explained to them.

### 2.2. Data Collection

Data were collected through a structured questionnaire directed to each child’s primary caregiver and through anthropometric and dental exams of each child performed by trained personnel in the family’s home. Initially, health community agents contacted the selected families to invite them to participate. Once the parent or legal guardian of a child signed the consent form, the health agent scheduled a visit with three members of the research team at the participants’ convenience.

A trained research assistant individually interviewed the primary home caregiver of each child and filled out a structured questionnaire. The questionnaire included questions about the child’s family (e.g., total family income, participation in the FHP with or without a dental team, the mother’s level of education). Additional questions regarding the age, gender, and oral health of the primary caregiver and the age, gender, and attendance at day care/school of each child were also included.

The weight and height of each child were measured according to World Health Organization (WHO) guidelines [33], and the values were recorded and analyzed using the nutritional survey model within WHO’s AnthroPlus v1.0.4 software. This software enables the calculation of growth reference data for children and adolescents (5–19 years) by generating Z-scores and percentile curves and cut-offs based on the standard deviations (SD) from the median. Children were categorized as severely thin (<−3SD), thin (<−2SD), normal weight (−2SD to +1SD), overweight (>+1SD, equivalent to BMI 25 kg/m^2^ at 19 years old), or obese (>+2SD, equivalent to BMI 30 kg/m^2^ at 19 years old) [34].

Following the height and weight measurements, a trained dentist from the FHP (examiner), who was assisted by a trained recorder, performed a dental exam to assess a child’s carious, missing or filled primary teeth (dmft index) according to the WHO guidelines [35]. The dentist sat in an ordinary chair and examined the child standing up in front of him, under natural light, with the help of an intraoral plane mirror and no probing. In addition to the dmft index, the Significant Caries Index (SiC) was also calculated. This value corresponds to the mean dmft of the subgroup of children with the highest caries scores (the top one-third of the sample) [36]. Children were also categorized as having or not having severe ECC (S-ECC), which was defined as a child having 6 or more primary teeth with caries.

Six dentists and 12 research assistants were trained in data collection by a professor who specializes in dental epidemiological surveys. The weight, height and dental status of a convenience sample of 13, 5-year-old children attending a day care center were determined. The inter-examiner and intra-examiner agreement for dental assessments were considered good (Kappa ranging from 0.6–0.8 and 0.6–1.0, respectively) [37]. A separate cohort of 20 adults accompanying children attending dental school clinics was interviewed to pilot the questionnaire.

### 2.3. Statistical Analysis

IBM SPSS Statistics Version 19 was used for statistical analyses. The primary outcome was dental caries, which were subdivided into the following three levels of severity: caries experience (dmft > 0), active ECC (6 > dmft > 0) and active S-ECC (dmft ≥ 6). The association between caries experience and each of the independent variables was evaluated. The independent variables examined included the gender and age of each child, the gender and age of each caregiver, each child’s nutritional status, the family income (in Brazilian minimum wages) and the mother’s formal education (years).

The statistical analyses included descriptive information and frequency distributions, bivariate analyses to test the associations between dental caries and BMI and logistic regression analysis (using the enter method) to evaluate the influence of the independent variables on caries experience (dichotomized as “yes” or “no”). Independent variables were entered into the regression analysis if they exhibited *p* values < 0.2 in the bivariate analysis. The level of significance was set as α ≤ 0.05.

## 3. Results

### 3.1. Participants

A total of 303 child-caregiver dyads were selected to participate in the present study. Of these, 29 participants were excluded because the parents did not provide consent, and five did not have the opportunity to have their weight or height assessed. Thus, 269 children with caregivers (respondent pairs) were studied. Of these, 238 (88.5%) were being seen by the FHP and 207 (77.0%) were being seen by an oral health team. The average monthly family income was equivalent to $668.77 (US dollars), and all homes had a fluoridated public water supply.

Caregivers were mostly mothers (n = 182, 67.7%), but grandparents also served as primary caregivers for some children (n = 53, 19.7%). Over one-third of the caregivers (n = 99, 36.8%) had not received any information about oral health in the previous months. Slightly more than half of the children were boys (n = 139, 51.7%), 75 were only children (27.9%) and 30 (11.1%) had three or more siblings. During the day, 172 children (63.9%) attended daycare centers or schools. According to the caregivers’ reports, only one child had a history of malnutrition. Most of the children (n = 171, 63.6%) had a history of frequent infections. Table 1 lists other characteristics of the participants.

**Table 1:** Sample characteristics.

Variable	Mean	Standard deviation	Minimum	Maximum
Family monthly income (as a fold-value compared to the average Brazilian minimum wage)	2.3	1.5	0.5	10.0
Mothers’ formal education (in years)	9.8	3.1	0	22.0
Caregivers’ age (in years)	35.3	12.0	12.0	69.0
Children’s age (in months)	68.7	3.8	61.0	79.0

### 3.2. Early Childhood Caries (ECC) and Body Mass Index (BMI)

**Figure 1:**
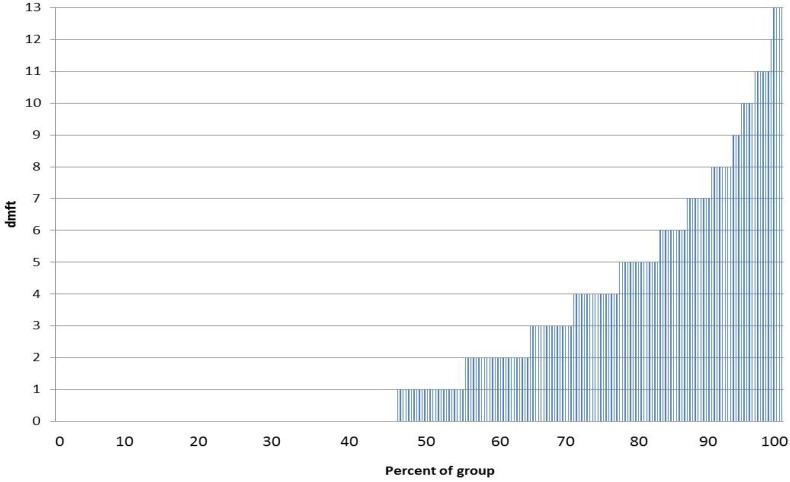
The cumulative distribution frequency of dental caries in this study population; 46.8% had no caries experience (decayed, missing, filled teeth = 0).

The dmft index was 2.5 ± 3.2 (mean ± SD) and ranged between 0 and 13. Decayed teeth were responsible for 68.4% of this index, whereas filled and missing teeth constituted 29.2% and 2.4% of this index, respectively. A majority of the children were caries-free at the time of the exam (n = 148 or 55.0%). The Significant Caries Index, which represents the occurrence of caries in a third of the analyzed sample, was high (SiC index = 6.4), thus indicating that the caries distribution in this sample is skewed (Figure 1). Among children with decayed teeth, 17.1% (n = 46) had six or more affected teeth and were diagnosed with S-ECC.

**Figure 2:**
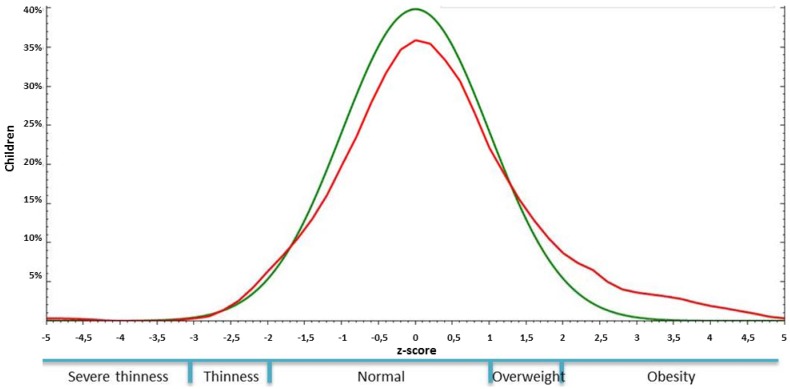
The Body Mass Index of children in the study. Data are shown as Z-scores (red line), and the WHO reference distribution (from 2007) for children 61 months to 19 years is also shown (green line).

The average BMI of the children in this study was 15.9 ± 2.2, and it ranged between 10.4 and 25.4. A total of 63 children were identified as overweight or obese (23.4%), which led to a left-skewed distribution compared to the WHO reference line (Figure 2). A majority of the children were of normal weight, but some children were either over or under the expected weight for their gender, age and height (Table 2). The frequencies of these BMI categories in children with or without caries, according to different criteria, are provided in Table 2.

**Table 2:** The frequency of BMI categories among children according to their dental caries status (*i.e.*, caries experience, active ECC or active S-ECC).

Body Mass Index Category ^(a)^	Caries experience, n (%)	Active Early Childhood Caries, n (%)	Active Severe Early Childhood Caries, n (%)
Severe thinness or thinness	1 (0.7%)	0 (0.0%)	0 (0.0%)
Adequate	114 (79.7%)	97 (80.2%)	39 (84.8%)
Overweight	18 (12.5%)	15 (12.4%)	5 (10.9%)
Obesity	10 (7.0%)	9 (7.4%)	2 (4.3%)
Total	143 (100.0%)	121 (100.0%)	46 (100.0%)

^(a)^ Body Mass Index categories according to Z-scores [34].

**Table 3:** Results showing the predictors of having caries experience.

Independent variables	OR	95%CI	*p*-value
Children being overweight or obese	1.32	0.70–2.50	0.40
Children’s age ^[1]^	0.94	0.88–1.00	0.06
Mother’s level of formal education ^[1]^	1.03	0.94–1.13	0.51
Family income ^[1]^	1.22	1.01–1.50	0.04

Nagelkerke R^2^ = 0.06; ^[1]^ Continuous variable.

Because there were only three cases of underweight children, we combined the underweight and normal weight BMI categories into a single group. Thus, being overweight was treated as a dichotomous variable (overweight or not), and the associations of these two categories with caries experience, ECC, and S-ECC (Chi-square) were evaluated. The *p*-values were above 0.05 for all three analyses. The logistic regression model analyzed “having no caries experience” as the main outcome, and the following four independent variables were included after the bivariate analysis: children being overweight or obese (*p* = 0.11, Chi-square test), children’s age (*p* = 0.05, Mann-Whitney test), mothers’ formal education (*p* = 0.02, Mann-Whitney test), and family income (*p* = 0.003, Mann-Whitney test). Higher family income was the only variable that was significantly associated with children having no caries experience, and this variable was able to correctly predict 61.8% of the cases with no caries experience (Nagelkerke R^2^ = 0.06) (Table 3).

## 4. Discussion

Nearly half of the children in this study had ECC, and approximately one-fourth were overweight or obese. However, several statistical analyses revealed no significant associations between these two variables. In contrast, the family income level was shown to be a significant predictor of children without ECC, but this factor only explained a modest amount of the variance. The relative weakness of the model was expected because only a small subset of the relevant variables was assessed. Indeed, the current conceptual model of ECC risk includes many social environmental, maternal, and childhood risk factors, including maternal psychological distress and dysfunctional parenting [38,39]. Nonetheless, our results showing a lack of a significant association between dental caries and BMI and the presence of a significant association between dental caries and socioeconomic condition are consistent with other studies conducted in children [14,18,19,23] and adolescents [40]. Because of the low numbers of underweight children in our sample, it was not possible to determine whether this subpopulation has a higher predisposition for caries [4,11,12,15,16]. The other studies that have shown a strong correlation between caries in primary dentitions and BMI did not control for social variables [9,17,21,24].

The influence of social health determinants on the incidence of caries is well known. A cross-sectional study of a subsample of 400 children nested in a Brazilian birth cohort revealed that the significant risk factors for caries in 6-year-old children were the mother’s educational status (less than 8 years of schooling), family income (less than six times the average minimum wage), pre-school non-attendance and the consumption of sweets at least once a day [41]. In another study that examined the FHP, the following risk factors were shown to be associated with ECC in 1,690 preschoolers aged between 18–36 months and 5 years old: a large number of household members, a lack of water supply, the length of time living in a place, the education level of the primary caregiver, public preschool attendance, the demand for oral health services and the pattern of sugar consumption [42]. It is likely that including additional social determinants in the current analysis (as the aforementioned studies did) would have produced a more robust model to explain the occurrence of ECC in our sample. However, the limitations of cross-sectional study designs should not be overlooked. For example, cross-sectional studies often miss many of the factors that influence a particular problem over time due to incomplete reporting and memory issues. These limitations can result in bias, especially when studying chronic diseases such as ECC and obesity. Indeed, the fact that a child is being served by a family health team at the time of the survey does not indicate that this child has always received this type of care. Similar arguments can hold true for other social and biological factors that can vary, such as family income. Thus, longitudinal studies may be more effective in developing a more realistic model for predicting ECC. Finally, although the lower limit of team agreement (kappa = 0.6) could be interpreted as reflecting a potential measurement error, we consider it to be a reliable parameter of moderate agreement for clinical measurements as cited by other authors [43,44].

Interestingly, longitudinal data from Swedish children have shown that while no significant association between the incidence of caries and obesity exists at 3 years of age, a significant association does emerge at 6 years of age and remains throughout adolescence and young adulthood [29]. Similarly, in another cohort with more than 2,300 Swedish children, the association between caries and obesity was weak, but it became significant when the children reached 12 years of age [26]. Based on these studies and the current findings, it can be hypothesized that because of the chronic nature of both caries and obesity, it is possible that young children (until at least approximately 6 years of age) have simply not been exposed to a family’s habits (e.g., diet) for a sufficient amount of time to have their BMI significantly affected. In agreement with this hypothesis, a study reported that although underweight 6-year-old children have more caries in primary dentitions, dental intervention did not significantly retard this increase in caries within 3 years. Furthermore, a study with a national American cohort reported that, in the preschool years, both favorable and unfavorable early weight statuses were highly associated with subsequent weight status at preschool [45]. Future studies are required to fully elucidate the effects of age on the relationship between obesity and caries.

Despite defining dental caries in several different ways, no statistically significant associations between dental caries and BMI were observed in the current sample of children. It is possible that an assessment of odontogenic infections would have yielded different results. Because dental treatment needs and pain occurrence were not investigated, it was not possible to determine the extent of decay. Another study with a population of Filipino adolescents showed that the PUFA index (pulp involvement, fistula, abscess) was significantly associated with low BMIs [46]. However, in a sample of patients with s-ECC, BMI was not correlated with dmft or the number of pulp-involved teeth [8].

The majority of families in this study were assisted by the FHP, but the duration of this assistance was not determined. This program has played a key role in improving maternal and child health in Brazil [47]. It has been shown that systematic home nutritional advice during the first year of life can decrease the incidence of dental caries by 22% in 4-year-old children [48]. Although a sample from two health districts of a large city in Brazil was studied, the results are likely generalizable because a stratified sample was selected and because dental caries and obesity are health conditions that are not spatially dependent [49].

Despite the limitations of this study, our results add to the literature investigating the association between ECC and obesity and provide additional evidence for the complexity of this relationship. Indeed, it appears likely that there is not a single common risk factor for these two disease processes but rather a complex interaction of health behaviors, social determinants and genetic factors that drive both caries and obesity. Regardless, we agree that child dental services and child welfare services can benefit from broader joint action [26].

## 5. Conclusions

No significant associations between dental caries and childhood obesity at 6 years of age were observed. However, lower family incomes were significant determinants of caries experience.

## References

[1] American Academy on Pediatric Dentistry (2008). Policy on early childhood caries (ECC): Classifications, consequences, and preventive strategies. Pediatr. Dent..

[2] Gaur S., Nayak R. (2011). Underweight in low socioeconomic status preschool children with severe early childhood caries. J. Indian Soc. Pedod. Prev. Dent..

[3] Gradella C.M., Bernabe E., Bonecker M., Oliveira L.B. (2011). Caries prevalence and severity, and quality of life in Brazilian 2- to 4-year-old children. Community Dent. Oral Epidemiol..

[4] Vania A., Parisella V., Capasso F., Di Tanna G.L., Vestri A., Ferrari M., Polimeni A. (2011). Early childhood caries underweight or overweight, that is the question. Eur. J. Paediatr. Dent..

[5] Caufield P.W., Li Y., Bromage T.G. (2012). Hypoplasia-associated severe early childhood caries—A proposed definition. J. Dent. Res..

[6] Kantovitz K.R., Pascon F.M., Rontani R.M., Gaviao M.B. (2006). Obesity and dental caries—A systematic review. Oral Health Prev. Dent..

[7] Werner S.L., Phillips C., Koroluk L.D. (2012). Association between childhood obesity and dental caries. Pediatr. Dent..

[8] Sheller B., Churchill S.S., Williams B.J., Davidson B. (2009). Body mass index of children with severe early childhood caries. Pediatr. Dent..

[9] Norberg C., Hallstrom Stalin U., Matsson L., Thorngren-Jerneck K., Klingberg G. (2012). Body mass index (BMI) and dental caries in 5-year-old children from southern Sweden. Community Dent. Oral Epidemiol..

[10] van Gemert-Schriks M.C., van Amerongen E.W., Aartman I.H., Wennink J.M., Ten Cate J.M., de Soet J.J. (2011). The influence of dental caries on body growth in prepubertal children. Clin. Oral Invest..

[11] Subramaniam P., Singh D. (2011). Association of age specific body mass index, dental caries and socioeconomic status of children and adolescents. J. Clin. Pediatr. Dent..

[12] Koksal E., Tekcicek M., Yalcin S.S., Tugrul B., Yalcin S., Pekcan G. (2011). Association between anthropometric measurements and dental caries in Turkish school children. Cent. Eur. J. Public Health.

[13] Ngoenwiwatkul Y., Leela-adisorn N. (2009). Effects of dental caries on nutritional status among first-grade primary school children. Asia Pac. J. Public Health.

[14] Floyd B. (2009). Associations between height, body mass, and frequency of decayed, extracted, and filled deciduous teeth among two cohorts of Taiwanese first graders. Am. J. Phys. Anthropol..

[15] Cameron F.L., Weaver L.T., Wright C.M., Welbury R.R. (2006). Dietary and social characteristics of children with severe tooth decay. Scott. Med. J..

[16] Oliveira L.B., Sheiham A., Bonecker M. (2008). Exploring the association of dental caries with social factors and nutritional status in Brazilian preschool children. Eur. J. Oral Sci..

[17] Trikaliotis A., Boka V., Kotsanos N., Karagiannis V., Hassapidou M. (2011). Short communication: Dmfs and BMI in preschool Greek children. An epidemiological study. Eur. Arch. Paediatr. Dent..

[18] Mojarad F., Maybodi M.H. (2011). Association between dental caries and body mass index among hamedan elementary school children in 2009. J. Dent. (Tehran).

[19] D’Mello G., Chia L., Hamilton S.D., Thomson W.M., Drummon B.K. (2011). Childhood obesity and dental caries among paediatric dental clinic attenders. Int. J. Paediatr. Dent..

[20] Costacurta M., Di Renzo L., Bianchi A., Fabiocchi F., De Lorenzo A., Docimo R. (2011). Obesity and dental caries in paediatric patients. A cross-sectional study. Eur. J. Paediatr. Dent..

[21] Vazquez-Nava F., Vazquez-Rodriguez E.M., Saldivar-Gonzalez A.H., Lin-Ochoa D., Martinez-Perales G.M., Joffre-Velazquez V.M. (2010). Association between obesity and dental caries in a group of preschool children in Mexico. J. Public Health Dent..

[22] Juarez-Lopez M.L., Villa-Ramos A. (2010). Caries prevalence in preschool children with overweight and obesity. Rev. Invest. Clin..

[23] Hong L., Ahmed A., McCunniff M., Overman P., Mathew M. (2008). Obesity and dental caries in children aged 2–6 years in the United States: National Health and Nutrition Examination Survey 1999–2002. J. Public Health Dent..

[24] Willershausen B., Moschos D., Azrak B., Blettner M. (2007). Correlation between oral health and body mass index (BMI) in 2071 primary school pupils. Eur. J. Med. Res..

[25] Marshall T.A., Eichenberger-Gilmore J.M., Broffitt B.A., Warren J.J., Levy S.M. (2007). Dental caries and childhood obesity: Roles of diet and socioeconomic status. Community Dent. Oral Epidemiol..

[26] Gerdin E.W., Angbratt M., Aronsson K., Eriksson E., Johansson I. (2008). Dental caries and body mass index by socio-economic status in Swedish children. Community Dent. Oral Epidemiol..

[27] Malek Mohammadi T., Wright C.M., Kay E.J. (2009). Childhood growth and dental caries. Community Dent. Health.

[28] Clarke M., Locker D., Berall G., Pencharz P., Kenny D.J., Judd P. (2006). Malnourishment in a population of young children with severe early childhood caries. Pediatr. Dent..

[29] Alm A., Isaksson H., Fahraeus C., Koch G., Andersson-Gare B., Nilsson M., Birkhed D., Wendts L.K. (2011). BMI status in Swedish children and young adults in relation to caries prevalence. Swed. Dent. J..

[30] Kopycka-Kedzierawski D.T., Auinger P., Billings R.J., Weitzman M. (2008). Caries status and overweight in 2- to 18-year-old US children: Findings from national surveys. Community Dent. Oral Epidemiol..

[31] Bonecker M., Ardenghi T.M., Oliveira L.B., Sheiham A., Marcenes W. (2010). Trends in dental caries in 1- to 4-year-old children in a Brazilian city between 1997 and 2008. Int. J. Paediatr. Dent..

[32] (2004). Projeto SB Brasil 2003: Condições de Saúde Bucal da População Brasileira 2002–2003: Resultados Principais.

[33] World Health Organization (1995). Expert Committee on Physical Status. Physical Status: The Use and Interpretation of Anthropometry.

[34] de Onis M., Onyango A.W., Borghi E., Siyam A., Nishida C., Siekmann J. (2007). Development of a WHO growth reference for school-aged children and adolescents. Bull. World Health Organ..

[35] World Health Organization (1997). Oral Health Surveys: Basic Methods.

[36] Bratthall D. (2000). Introducing the Significant Caries Index together with a proposal for a new global oral health goal for 12-year-olds. Int. Dent. J..

[37] Cicchetti D.V., Sparrow S.A. (1981). Developing criteria for establishing interrater reliability of specific items: Applications to assessment of adaptive behavior. Am. J. Ment. Defic..

[38] Kim Seow W. (2012). Environmental, maternal, and child factors which contribute to early childhood caries: A unifying conceptual model. Int. J. Paediatr. Dent..

[39] Hooley M., Skouteris H., Boganin C., Satur J., Kilpatrick N. (2012). Parental influence and the development of dental caries in children aged 0–6 years: A systematic review of the literature. J. Dent..

[40] Sales-Peres S.H., Goya S., Sant’Anna R.M., Silva H.M., Sales-Peres Ade C., Silva R.P., Lauris J.R., Bastos J.R. (2010). [Prevalence of overweight and obesity, and associated factors in adolescents, at the central west area of the state Sao Paulo (SP, Brazil)]. Cien. Saude Colet..

[41] Peres M.A., Latorre M.R., Sheiham A., Peres K.G., Barros F.C., Hernandez P.G., Maas A.M.N., Romano A.R., Victora C.G. (2003). Effects of social and biological factors on dental caries in 6-year-old children: A cross sectional study nested in a birth cohort in Southern Brazil. Rev. Bras. Epidemiol..

[42] Melo M.M., Souza W.V., Lima M.L., Braga C. (2011). Factors associated with dental caries in preschoolers in Recife, Pernambuco State, Brazil. Cad. Saude Publica.

[43] Landis J.R., Koch G.G. (1977). The measurement of observer agreement for categorical data. Biometrics.

[44] Vieira A.J., Garret J.M. (2005). Understanding interobserver agreement: The kappa statistic. Fam. Med..

[45] Moss B.G., Yeaton W.H. (2012). U.S. children’s preschool weight status trajectories: Patterns from 9-month, 2-year, and 4-year Early Childhood Longitudinal Study—Birth cohort data. Am. J. Health Promot..

[46] Benzian H., Monse B., Heinrich-Weltzien R., Hobdell M., Mulder J., van Palenstein Helderman W. (2011). Untreated severe dental decay: A neglected determinant of low Body Mass Index in 12-year-old Filipino children. BMC Public Health.

[47] Victora C.G., Aquino E.M., do Carmo Leal M., Monteiro C.A., Barros F.C., Szwarcwald C.L. (2011). Maternal and child health in Brazil: Progress and challenges. Lancet.

[48] Feldens C.A., Giugliani E.R., Duncan B.B., Drachler Mde L., Vitolo M.R. (2010). Long-term effectiveness of a nutritional program in reducing early childhood caries: A randomized trial. Community Dent. Oral Epidemiol..

[49] Campos J.A., Melanda E.A., Antunes Jda S., Foschini A.L. (2011). Dental caries and the nutritional status of preschool children: A spatial analysis. Cien. Saude Colet..

